# Surgical treatment of male breast cancer metastasis to thoracic spine: A case report

**DOI:** 10.1097/MD.0000000000036252

**Published:** 2023-12-08

**Authors:** Jong-Hyun Ko, Jong-Hong Kim, Dong-Hun Ham

**Affiliations:** a Department of Orthopedic Surgery, Research Institute of Clinical Medicine of Jeonbuk National University – Biomedical Research Institute of Jeonbuk National University Hospital, Jeonju, Jeonbuk, Republic of Korea; b Department of Orthopedic Surgery, Jeonbuk National University Hospital, Jeonju, Jeonbuk, Republic of Korea; c Department of Orthopedic Surgery, St. Carollo General Hospital, Suncheon, Jeonnam, Republic of Korea.

**Keywords:** corpectomy, male breast cancer, separation surgery, spine metastasis

## Abstract

**Purpose:**

We present a rare clinical case of a metastatic spinal tumor in the 7th thoracic spine from male breast cancer (MBC).

**Method:**

A 62-year-old man was referred as an outpatient, complaining of continuous pain in the back and right flank that began 2 weeks earlier. The patient had no neurologic signs or symptoms but had a medical history of left breast modified radical mastectomy because of MBC. Computed tomography and magnetic resonance imaging showed metastasis in the T7 vertebra and no other metastasis on positron emission tomography/computed tomography or bone scan. Separation surgery was performed with posterior corpectomy of T7 (en bloc excision), followed by stabilization with an expandable titanium cage and pedicle screws. The pathological examination of the excised T7 vertebra confirmed metastatic carcinoma with neuroendocrine differentiation from the breast. Adjuvant chemo-radiotherapy was performed after surgery.

**Results:**

The patient had no symptoms at the 21-month follow-up. Radiologic studies showed no evidence of recurrent or metastatic lesions.

**Conclusion:**

MBC is extremely rare, with fewer cases of spinal metastases. Among these, patients who undergo separation surgery are even rarer. This case shows that radical surgery can be an option for MBC with spine metastasis if indicated.

## 1. Introduction

Breast cancer is a rare entity, with an incidence of 1 in 100,000 male patients. Bone is the most common site for secondary deposits from breast cancer.^[1]^ As the survival time of cancer patients increases, the diagnosis of metastatic spinal tumors is also increasing.^[2]^ Spinal involvement has complications of pathological fractures, instability, cord compression, and resulting intractable pain, paralysis, and incontinence. Reports of male patients with breast cancer complicated by spinal metastases are very few, and this report is the 11th.^[3]^

Although male breast cancer (MBC) is clinically and epidemiologically similar to female breast cancer, and its treatment follows the same indications, the survival rates are significantly lower.^[4]^ We present a case of MBC metastasis to the thoracic spine, treated by separation and stabilization of the lesion, followed by adjuvant chemo-radiotherapy. To our knowledge, no case in which a corpectomy was performed has been reported.

### 1.1. Consent

The patient signed informed consent for the publication of this case report and any accompanying images. Ethical approval of this study was waived by the Ethics Committee of Jeonbuk National University Hospital because it was a case report and there were fewer than 3 patients.

## 2. Case presentation

**Figure 1.**
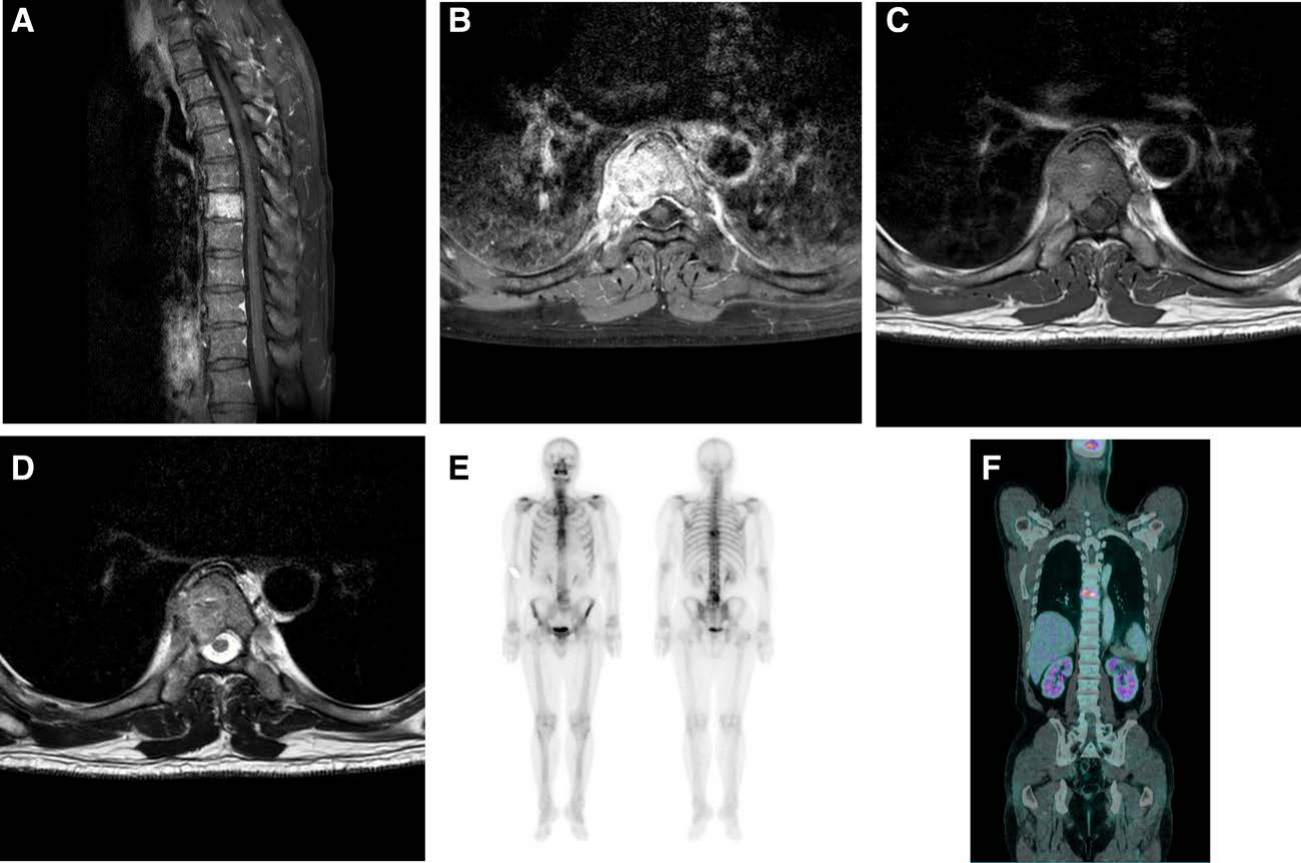
Magnetic resonance imaging (MRI): A and B are coronal and axial T1-weighted images after gadolinium administration, showing neoplastic involvement of the T7 vertebral body. C and D show grade II epidural spinal cord compression. Bone scan and PET CT: E and F show T7 involvement. PET CT = positron emission tomography/computed tomography.

A 62-year-old male was referred to our orthopedic outpatient department because of a metastatic spinal tumor. Upon hospitalization, he presented with mechanical back pain in the thoracic spine without any other neurological signs or symptoms. He had undergone total modified radical mastectomy with axillary lymphadenectomy one and half years earlier for MBC. Because of this, magnetic resonance imaging, bone scan, and positron emission tomography/computed tomography were performed and pathological masses at the T7 level, attributable to breast cancer metastases, were found. Total body positron emission tomography/computed tomography and bone scan revealed no other metastases (Fig. 1).

**Figure 2.**
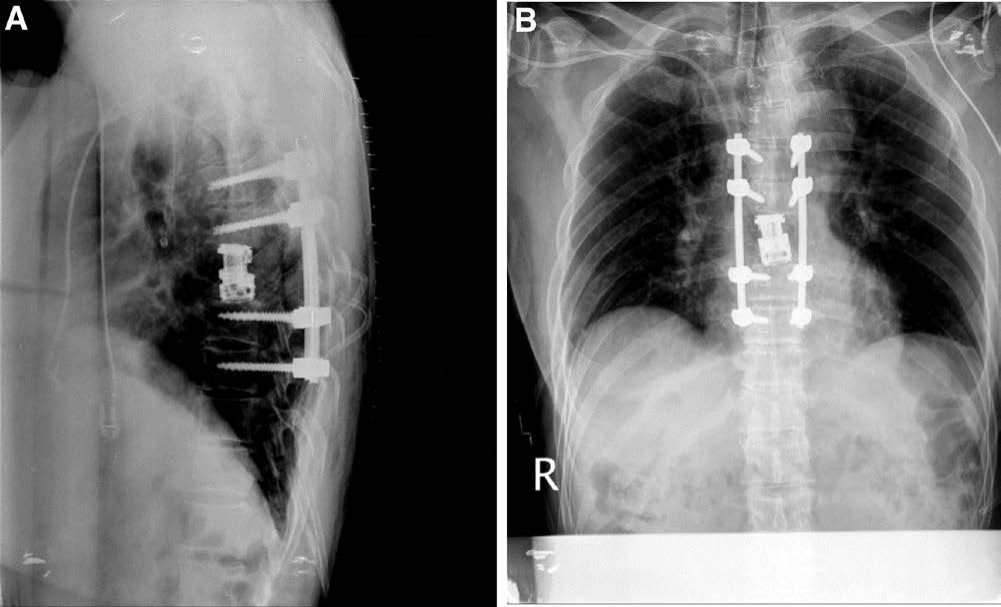
Radiographs: A and B show the excision of the T7 vertebral body with an expandable cage and posterior stabilization with pedicle screws and rods.

**Figure 3.**
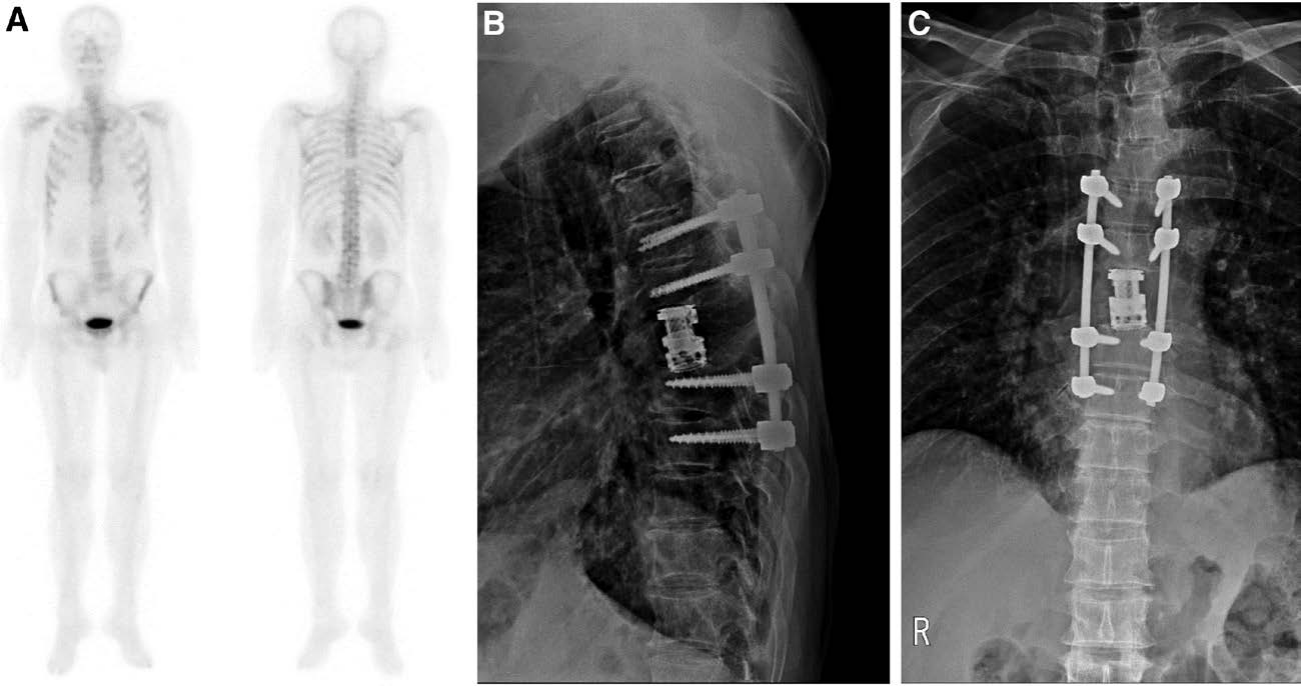
Latest follow-up bone scan (POD 20 mo): A showing no metastasis. Latest follow-up radiographs (POD 18 mo): B and C show well-maintained stabilization.

After a multidisciplinary consult, surgical radical resection consisting of en bloc corpectomy based on multiple treatment guidelines was decided. During surgical treatment, the patient underwent T7 corpectomy by the posterior approach and expandable cage insertion (Stratosphere expandable corpectomy system) followed by screw and rod fixation (T5–T9) (Fig. 2). The right T7 pedicle seemed to have been invaded by a metastatic tumor, and a biopsy was performed along with vertebral body and intervertebral discs. The patient was transferred to a general ward after a day of intensive unit care for postoperative closed monitoring. The patient was discharged 2 weeks after surgery without complications. A biopsy performed on the vertebral body ultimately confirmed metastatic carcinoma, most likely from the breast. Cycles of adjuvant 3D chemo-radiotherapy followed. The patient is still alive 21 months later and in good general condition without metastasis (Fig. 3).

The patient scored 80 points or more on the Karnofsky performance status scale, the global performance status of patients with spinal metastases was fair or excellent in the initial assessment algorithm, and his life expectancy was expected to be more than 2 months. For a more precise systemic assessment, the life expectancy was predicted to be more than 1 year from a Tomita score of 2 and a modified Tokuhashi score of 13.^[5,6]^ After modified radical mastectomy, a single lesion had metastasized to the thoracic spine. Systemic disease was thought to be progressing, but effective systemic treatment was judged to be available. Therefore, the MNOP algorithm could be applied.^[7]^

According to the MNOP algorithm and the NOMS framework, our case was epidural spinal cord compression grade II, as the neurologic evaluation showed spinal cord compression and cerebrospinal fluid was visible. In the oncologic assessment, according to Spratt et al (2017), in the radiosensitivity category, breast cancer goes under intermediate grades of effect of tumor histology on conventional EBRT outcomes. In mechanical assessment, the patient had indeterminate stability with 8 SINS score points. In systemic assessment, the patient was in a systemic condition with no specific past history, there was no difficulty in undergoing surgery, and life expectancy was predicted to be more than 1 year with a Tomita score of 2 and a modified Tokuhashi score of 13. According to the MNOP algorithm for spinal metastasis management, conventional external beam radiation therapy (cEBRT) or separation surgery followed by cEBRT or stereostatic radiosurgery (SRS) could be treatments of choice.^[7,8]^

Thus, we decided to perform adjuvant radiation therapy after separation and stabilization surgery for the following reasons. First, the patient was in good general condition. Second, he had only a single focal metastasis, and third, there was cord deformation in the neurologic assessment, and separation was performed to prevent possible neurologic deficits in the future. Therefore, it was deemed necessary. The surgery went well, as planned. Ten cycles of cEBRT were performed after wound management was completed, and the patient has had no major problems so far.

## 3. Discussion

The origin of cancers in global metastatic spinal tumors is most frequently the breast (18.5%), prostate (14.8%), lung (13.9%), kidney (12%), and myeloma (5.9%). In Asia, the order of occurrence is the lung (28.1%), liver (13.2%), kidney (10.9%), colon (6.4%), and breast (5.9%).^[6–9]^ Skeletal involvement is very frequent in patients with MBC (an incidence of 47%–85% in autopsy series). Despite the high incidence of BC metastasis to the spine, very few clinical reports have dealt specifically with MBC metastases.^[4]^

MBC patients have a very short life expectancy from diagnosis. With conventional treatment, most patients receive palliative treatment rather than radical surgery. According to the latest knowledge, using the algorithm for patients with spinal metastases, more than supportive care is necessary, even if the life expectancy is 2 months or more.^[7]^

To our knowledge, about ten cases of metastatic spinal MBC have been reported in the literature, and operative treatment was reported in only 2 cases. In 1 case, the patient underwent C3 vertebroplasty because of cervicalgia. In the other case, the patient underwent T8 decompression and stabilization of T7 to T9.^[4,10]^ Thus, patients who underwent separation are even rarer.

The clinical outcome of metastatic spinal tumor patients has improved with the development of SRS and cEBRT, but the treatment decision itself has become more complicated with the development of these therapies.^[11]^ Metastatic spine tumors are challenging for both patients and spine surgeons since it is difficult to determine a consistent treatment policy depending on each patient socioeconomic status, type of primary cancer, metastasis site, and other factors. However, now it is generally accepted to perform radiotherapy (cEBRT or SRS) based on mechanical stability, neurologic risk, and oncological parameters, or surgical treatment and adjuvant radiotherapy.

Determining a treatment method for isolated MBC metastasis is not an easy process due to its rarity, and some spine surgeons may prefer a more conservative treatment, such as radiotherapy, rather than surgical treatment. However, the good results achieved in this case based on the MNOP algorithm are thought to have implications for determining treatment policies for isolated metastasis in relatively active males.

## Author contributions

**Conceptualization:** Jong-Hyun Ko.

**Data curation:** Jong-Hong Kim.

**Investigation:** Dong-Hun Ham.

**Writing – original draft:** Jong-Hong Kim.

**Writing – review & editing:** Jong-Hyun Ko, Dong-Hun Ham.
